# Single Nucleotide Polymorphisms in *HOTAIR* Are Related to Breast Cancer Risk and Prognosis in the Northeastern Chinese Population

**DOI:** 10.3389/fonc.2021.706428

**Published:** 2021-07-12

**Authors:** Zheng Lv, Changgui Kou, Naifei Chen, Lin Jia, Xu Sun, Yangyang Gao, Rilan Bai, Ming Yang, Jiuwei Cui

**Affiliations:** ^1^ Cancer Center, The First Hospital of Jilin University, Changchun, China; ^2^ Department of Epidemiology and Biostatistics, School of Public Health, Jilin University, Changchun, China; ^3^ Shandong Provincial Key Laboratory of Radiation Oncology, Cancer Research Center, Shandong, Cancer Hospital and Institute, Shandong First Medical University and Shandong Academy of Medical Sciences, Jinan, China

**Keywords:** *HOTAIR*, breast cancer, susceptibility, prognosis, single nucleotide polymorphisms

## Abstract

**Background:**

The long noncoding RNA HOX transcript antisense RNA (*HOTAIR*) is highly expressed in breast cancer (BC) tissues and is associated with the recurrence and metastasis of BC. Until now, the results of studies on associations between several functional single nucleotide polymorphisms(SNPs) (rs920778, rs1899663, and rs4759314) in *HOTAIR* with BC susceptibility carried out in different regions of China are still inconsistent. There is no study on correlation between *HOTAIR* SNPs and prognosis of Chinese population. Therefore, we investigated the relationship between *HOTAIR* SNPs and susceptibility to and prognosis of BC.

**Method:**

We conducted a population-based case-control study involving 828 BC cases and 905 healthy controls. Peripheral blood DNA was used for genotyping. The association between *HOTAIR* genotypes and BC risk were estimated by odds ratios (ORs) computed using the binary logistic regression model. The relationships between *HOTAIR* SNPs and clinicopathological features were tested by Pearson’s chi-square test or Fisher’s exact test. Survival was analyzed using the Kaplan-Meier method.

**Results:**

The functional rs920778 genetic variant increased BC risk in the codominant model. Individuals with the rs920778 GG genotype had an OR of 2.426 (95% confidence interval [CI] = 1.491–3.947, P < 0.001) for developing BC compared to individuals with the AA genotype. Individuals with the AG genotype had an OR of 1.296 (95% CI = 1.040–1.614, P = 0.021) for developing BC compared to individuals with the AA genotype. Individuals with the rs4759314 GA genotype had a lower BC risk than individuals with the rs4759314 AA/GG genotype (OR = 0.566, 95% CI = 0.398–0.803, P = 0.001). The rs1899663 genotype had no correlation with BC susceptibility. Haplotypes composed of rs920778–rs1899663 and rs920778–rs1899663–rs4759314 could increase BC risk (all P < 0.001). There were no statistically significant associations between *HOTAIR* SNPs and clinicopathological characteristics. The rs920778 GG/AG genotypes were associated with worse disease-free survival (DFS) (p = 0.012), and the rs4759314 GA genotype was associated with worse DFS and overall survival (OS) (p = 0.011).

**Conclusion:**

*HOTAIR* SNPs(rs920778 and rs4759314) are significantly related to BC susceptibility and prognosis in the northeastern Chinese population, indicating the significance in the occurrence and development of BC.

## Introduction

Breast cancer (BC) is one of the most common cancers among women, and its morbidity and mortality have continued to increase worldwide in recent years, reflecting its strong invasive and metastatic characteristics (1, 2). In China, the incidence of BC is increasing annually and is currently the most common malignant tumor in women (3, 4).

Long noncoding RNAs are non-protein-coding transcripts longer than 200 nt and play important roles in the epigenetic regulation of gene expression. One such RNA, HOX transcript antisense RNA (*HOTAIR*), is transcribed from the antisense strand of the *HOXC* locus and mainly regulates *HOXD* genes. *HOTAIR* can guide the polycomb repressor complex 2/lysine-specific histone demethylase 1 complex to a specific target gene, where the complex then trimethylates lysine 27 of histone H3 and dimethylates lysine 4 of histone H3, causing chromatin remodeling (5–7). This can block some metastasis suppressor genes, such as junctional adhesion molecule 2, protocadherin beta 5, and protocadherin 10 (6).

*HOTAIR* is overexpressed in BC and is related to the occurrence, development, recurrence, and metastasis of BC. A large number of researches indicate that *HOTAIR* has oncogenic impacts. In the diagnosis of gastric cancer, pancreatic cancer, and colorectal cancer, the expression of *HOTAIR* is used to distinguish benign and malignant tissues, compared with benign tissues, the expression of *HOTAIR* in tumor tissues is higher. *HOTAIR* is a biomarker of therapeutic response and poor prognosis (8). In our previous studies, we identified several single nucleotide polymorphisms (SNPs) in *HOTAIR* (rs920778, rs4759314, and rs1899663). These SNPs are located in the intronic region of *HOTAIR* and can regulate its expression (9–11). Therefore, these SNPs are expected to be related to the occurrence, development, recurrence, and metastasis of BC. These SNPs may have the potential to be a new therapeutic target. Further research demonstrated that these sites are related to gastric cancer, esophageal cancer, and papillary thyroid cancer susceptibility. Several meta-analyses showed that these SNPs are associated with the susceptibility of gastrointestinal cancer and estrogen-dependent tumors (12–17), especially in Asian populations. However, these SNPs have different prevalences in different regions and races and are more common in Asian populations than in Caucasian populations. There are also different prevalences in different parts of Asia (12, 17). Few studies have reported a relationship between *HOTAIR* SNPs and BC susceptibility. The participants of the current study were mainly Chinese, Turkish, Iranian, and Indian. The results of the research on populations in different regions are inconsistent and controversial. There are obvious regional differences in the distribution of *HOTAIR* genetic polymorphisms in gastrointestinal cancer. The GG genotype of rs920778 in northeastern population is higher than in middle or southern population, the GG genotype of rs4759314 in southeastern population is higher than in middle and northern population, the GG genotype of rs1899663 in southeastern population is lower than in middle and northern population. Therefore, it is of great significance for us to study the role of *HOTAIR* gene polymorphisms in the occurrence, development, and prognosis of BC in the Northeast population for the first time. This can provide research basis for discovering new pathogenic targets of BC.Therefore, we retrospectively analyzed the relationship between *HOTAIR* SNPs (rs920778, rs1899663, and rs4759314) and BC clinicopathological features and prognosis in the northeastern Chinese population.

## Materials and Methods

### Ethics

This study was approved by the Institutional Ethics Committee of our hospital (ethical approval number 2014-031). Written informed consent was obtained from each participant at recruitment. The study methods were carried out in accordance with the relevant guidelines.

### Study Design

#### Selection and Description of Participants

We investigated the relationship between *HOTAIR* SNPs (rs920778, rs1899663, and rs4759314) and the risk of BC in a case-control study. All of the participants were genetically unrelated Han Chinese individuals from northeast China. This study enrolled 828 BC patients and 905 age-matched healthy control individuals from The First Affiliated Hospital of Jilin University (Changchun, Jilin Province, China) between April 2013 and September 2016. The median follow-up time was 6.7 years. The participants’ clinical characteristics were collected through medical records. The inclusion criteria were female patients with early breast cancer diagnosed by pathology.

#### *HOTAIR* SNP Genotyping

DNA was extracted from peripheral blood samples. Genotypes were detected using the MassArray system (Agena, San Diego, CA, USA) by the matrix-assisted laser desorption ionization-time of flight mass spectrometry method. *HOTAIR* was selected and genotyped as described previously (9–11). SNP genotyping was performed without knowledge of case status. Reciprocal testing was performed in a random sample of 15%, and the reproducibility was 99.7%.

### Statistics

SPSS 24.0 (IBM Corp., Armonk, NY, USA) and the online SNPStats program (https://www.snpstats.net/start.htm, developed by the Institut Català d’Oncologia) were used to analyze BC risk. Variables are characterized as percentages. The Hardy-Weinberg equilibrium test was conducted to test whether the allele frequency distribution of the case group and the control group is biased. Pearson’s chi-square test was used to examine differences in demographic variables and *HOTAIR* htSNP genotype distributions between BC cases and controls. Associations between *HOTAIR* genotypes and BC risk were estimated by odds ratios (ORs) and their 95% confidence intervals (CIs), which were computed using the binary logistic regression model. All ORs were adjusted by age whenever appropriate. Pearson’s chi-square test or Fisher’s exact test were used to evaluate the relationships between *HOTAIR* SNPs and clinicopathological features. The effects of the *HOTAIR* SNPs on disease-free survival (DFS) and overall survival (OS) were evaluated using the Kaplan-Meier method and the univariate Cox model. All statistical tests were two-sided. P values < 0.05 were considered statistically significant.

## Results

### Participant Characteristics

The control group was composed of healthy women who had undergone routine physical examination in our hospital who did not have a family history of cancer. The median age of the control group was 38 years (range 32–53 years). There were 678 premenopausal women and 226 postmenopausal women. The median age of the case group was 51 years (range 44–58 years), in which there were 398 premenopausal women and 430 postmenopausal women. Only 32 cases had a family history of cancer. Among 828 BC cases, 793 were of an invasive ductal carcinoma and 35 were of other types. Detailed information on the characteristics of the BC patients can be found in **Table 1**.

**Table 1:** Clinical characteristics of breast cancer patients.

Characteristics	Cases No. (%)
Median age (years)	51 (44-58)
Menstrual status	
Premenopause	398 (48.07)
Postmenopause	430 (51.93)
Family history	
Negative	796 (96.14)
Positive	32 (3.86)
Pathological type	
Invasive ductal carcinoma	793 (95.77)
Other types	35 (4.23)
Histological grade	
I	31 (3.74)
II	511 (61.71)
III	286 (34.54)
Tumor size	
T1	422(50.97)
T2	365(44.08)
T3	27(3.26)
T4	14(1.69)
Lymph node	
N0	396 (47.83)
N1	285 (34.42)
N2	101 (12.20)
N3	46 (5.58)
Lymphovascular invasion	
Negative	471 (56.88)
positive	357 (43.12)
Total	828 (100.00)

### Relationship Between *HOTAIR* SNPs and Risk of BC

The genotype distribution of cases and controls showed no deviation for different *HOTAIR* SNPs either in controls or in cases (**Table 2**). The functional rs920778 genetic variant was associated with an increased risk of BC in three genetic models. We used the Akaike Information Criterion to select the optimal genetic model, and the lowest AIC was found in the codominant genetic model. We discovered that the rs920778 GG genotype had an OR for BC development of 2.426 (95% CI = 1.491–3.947, P < 0.001) compared to the AA genotype. The rs920778 AG genotype was also associated with an increased BC risk compared to the rs920778 AA genotype (OR = 1.296, 95% CI = 1.040–1.614, P = 0.021). The functional rs4759314 genetic variants had different associations with BC risk in different genetic models (i.e., the codominant model, dominant model, and overdominant model). The AIC was the lowest in the overdominant model; therefore, using that model, the rs4759314 GA genotype was associated with a lower risk of BC development (OR = 0.566, 95% CI = 0.398–0.803, P = 0.001) than the AA/GG genotype. The rs1899663 SNP did not show an association with BC risk (**Table 3**).

**Table 2:** Hardy-Weinberg equilibrium test for different *HOTAIR* SNPs.

SNPs	Cases				Controls			
	^1^H_0_	^1^He	χ^2^	*P*	^1^H_0_	^1^He	χ^2^	*P*
rs920778	0.3321	0.3545	3.2952	0.0695	0.3105	0.3072	0.1054	0.7455
rs1899663	0.2923	0.3043	1.2960	0.2549	0.3127	0.2989	1.9426	0.1634
rs4759314	0.0743	0.0870	17.5992	<0.001	0.1149	0.1142	0.0398	0.8419

^1^H0:observed value of heterozygote frequency; ^1^He:expected value of heterozygote frequency.

**Table 3:** Association between *HOTAIR* SNPs and breast cancer risk.

SNP	Genotype	Model	Cases No.(%)	Controls No.(%)	OR (95%CI)^a^	*P* value	AIC
rs920778	AA	Codominant	498(60.36)	593(65.52)	1.000	<0.001	2174.4
	AG		274(33.21)	281(31.05)	1.296(1.040-1.614)	0.021	
	GG		53(6.43)	31(3.43)	2.426(1.491-3.947)	<0.001	
	AA	Dominant	498(60.36)	593(65.52)	1.000	0.001	2180.6
	AG/GG		327(39.64)	312(34.48)	1.406(1.140-1.735)		
	AA/AG	Recessive	772(93.58)	874(96.57)	1.000	0.001	2177.2
	GG		53(6.42)	31(3.43)	2.220(1.373-3.588)		
	AA/GG	Overdominant	551(66.79)	624(68.95)	1.000	0.078	2188.1
	AG		274(33.21)	281(31.05)	1.215(0.979-1.509)		
rs1899663	CC	Codominant	552(66.67)	598(66.08)	1.000	0.163	2190.6
	CA		242(29.23)	283(31.27)	0.910(0.729-1.134)	0.400	
	AA		34(4.10)	24(2.65)	1.586(0.900-2.793)	0.111	
	CC	Dominant	552(66.67)	598(66.08)	1.000	0.721	2194.3
	CA/AA		276(33.33)	307(33.92)	0.962(0.778-1.190)		
	CC/CA	Recessive	794(95.89)	881(97.35)	1.000	0.087	2190.1
	AA		34(4.11)	24(2.65)	1.633(0.931-2.864)		
	CC/AA	Overdominant	586(70.77)	622(68.73)	1.000	0.296	2192.6
	CA		242(29.23)	283(31.27)	0.890(0.715-1.108)		
rs4759314	AA	Codominant	756(91.64)	798(88.18)	1.000	0.004	2182.8
	GA		62(7.52)	104(11.49)	0.568(0.400-0.807)	0.002	
	GG		7(0.84)	3(0.33)	1.930(0.459-8.119)	0.370	
	AA	Dominant	756(91.64)	798(88.18)	1.000	0.004	2184
	GA/GG		69(8.36)	107(11.82)	0.609(0.434-0.855)		
	AA/GA	Recessive	818(99.15)	902(99.67)	1.000	0.331	2189.3
	GG		7(0.85)	3(0.33)	2.039(0.486-8.560)		
	AA/GG	Overdominant	763(92.48)	801(88.51)	1.000	0.001	2182
	GA		62(7.52)	104(11.49)	0.566(0.398-0.803)		

a OR and 95%CI were analyzed by logistic regression and adjusted by age. Common genotype was taken as reference.

### Haplotype Analysis

In order to analyze the influence of different haplotype systems composed of three *HOTAIR* SNP sites on the occurrence of BC, We explored the correlation between haplotypes and BC risk by comparing the distribution of each haplotype in the case group and the control group. There were significant differences between the case and control groups in the distributions of the following haplotypes: rs920778–rs1899663 and rs920778–rs1899663–rs4759314 (all P < 0.001). However, rs1899663–rs4759314 was not related to BC risk (**Table 4**). Haplotype 1 is composed of wild-type genotypes of three SNPs. Haplotype 2 increased BC risk compared with haplotype 1 (OR=1.39, 95%CI=1.13-1.70, P=0.002). Haplotype 4,5, and 6 reduced BC risk compared with haplotype 1 (all P < 0.001) (**Table 5**).

**Table 4:** Association between haplotypes in *HOTAIR* and breast cancer risk.

Haplotypes	df	Global P
rs920778-rs1899663	3	<0.001
rs1899663-rs4759314	3	0.100
rs920778-rs1899663-rs4759314	7	<0.001

adjusted by age.

**Table 5:** Haplotype distribution analysis.

Haplotype	rs920778	rs1899663	rs4759314	Controls: Case frequency		*OR*(95%*CI*)	*p*
1				0.7353:	0.7657	1.00	
2	G	A	A	0.1313:	0.1854	1.39(1.13-1.70)	0.002
3	G	C	G	0.0354:	0.0453	1.32(0.91-1.92)	0.140
4	A	A	A	0.0498:	0.0018	0.02(1.01-0.07)	<0.001
5	A	C	G	0.0237:	0.0006	0.01(0.00-0.10)	<0.001
6	G	C	A	0.0228:	0.0012	0.03(0.01-0.12)	<0.001
rare	A	A	G	0.0017:	NA	0.00(-)	–

### Relationship Between *HOTAIR* SNPs and Prognosis of BC

We did not find any significant associations between *HOTAIR* SNPs and clinicopathological characteristics of BC, including tumor size, lymph node metastasis, lymphovascular invasion, molecular type, histological grade, family history, menstrual status, and pathological type (**Table 6**). We then assessed the correlation between *HOTAIR* SNPs and survival in Cox regression analysis. GA genotype of rs920778 and GA genotype of rs4759314 could predict poor prognosis both in univariate analysis and multivariate analysis (**Tables 7** and **8**).

**Table 6:** Association between *HOTAIR* SNPs and BC clinical characteristics.

Characteristic	Grouping	rs920778 genotype			P	rs4759314 genotype			P	rs1899663 genotype			P
		AA n(%)	GA n(%)	GG n(%)		AA n(%)	GA n(%)	GG n(%)		AA n(%)	GAn(%)	GG n(%)	
**Molecular type**	**luminalA**	62 (12.45)	37 (13.50)	5 (9.44)	0.650	95 (12.57)	8 (12.90)	1 (14.29)	0.649	70 (12.68)	30 (12.40)	4 (11.76)	0.527
	**luminalB**	324 (65.06)	161 (58.76)	33 (62.26)		473 (62.57)	42 (67.74)	3 (42.86)		358 (64.86)	142 (58.68)	20 (58.82)	
	**HER2**	58 (11.65)	38 (13.87)	7 (13.21)		94 (12.43)	7 (11.29)	1 (14.29)		65 (11.78)	34 (14.05)	4 (11.76)	
	**Triple negative**	54 (10.84)	38 (13.87)	8 (15.09)		94 (12.43)	5 (8.07)	2 (28.56)		5 9(10.68)	36 (14.87)	6 (17.66)	
**Lymph node**	**N0**	251 (50.40)	120 (43.80)	23 (43.40)	0.258	363 (48.02)	25 (40.32)	6 (85.71)	0.200	273 (49.46)	111 (45.87)	12 (35.29)	0.407
	**N1**	168 (33.73)	94 (34.31)	22 (41.51)		260 (34.39)	24 (38.71)	0 (0.00)		187 (33.88)	82 (33.88)	16 (47.06)	
	**N2**	53 (10.64)	41 (14.96)	7 (13.21)		93 (12.30)	7 (11.29)	1 (14.29)		60 (10.86)	36 (14.88)	5 (14.71)	
	**N3**	26 (5.23)	19 (6.93)	1 (1.88)		40 (5.29)	6 (9.68)	0 (0.00)		32 (5.80)	13 (5.37)	1 (2.94)	
**Tumor size**	**T1**	264 (53.01)	137 (50.00)	19 (35.85)	0.221	390 (51.59)	30 (48.39)	1 (14.29)	0.397	288 (52.17)	119 (49.17)	15 (44.12)	0.505
	**T2**	213 (42.77)	121 (44.16)	31 (58.49)		327 (43.25)	30 (48.39)	6 (85.71)		240 (43.48)	109 (45.04)	16 (47.06)	
	**T3**	14 (2.81)	11 (4.01)	1 (1.89)		26 (3.44)	1 (1.61)	0 (0.00)		16 (2.90)	10 (4.13)	1 (2.94)	
	**T4**	7 (1.41)	5 (1.83)	2 (3.77)		13 (1.72)	1 (1.61)	0 (0.00)		8 (1.45)	4 (1.66)	2 (5.88)	
**Menstrual status**	**Premenopause**	244 (49.00)	124 (45.26)	29 (54.72)	0.372	359 (47.49)	34 (54.84)	4 (57.14)	0.475	276 (50.00)	102 (42.15)	20 (58.82)	0.055
	**Postmenopause**	254 (51.00)	150 (54.74)	24 (45.28)		397 (52.51)	28 (45.16)	3 (42.86)		276 (50.00)	140 (57.85)	14 (41.18)	
**Family history**	**negative**	474 (95.18)	266 (97.08)	53 (100.00)	0.136	726 (96.03)	60 (96.77)	7 b(100.00)	1.000	526 (95.29)	236 (97.52)	34 (100.00)	0.159
	**positive**	24 (4.82)	8 (2.92)	0 (0.00)		30 (3.97)	2 (3.23)	0 (0.00)		26 (4.71)	6 (2.48)	0 (0.00)	
**Histological grade**	**Ⅰ**	20 (4.02)	8 (2.92)	3 (5.66)	0.877	28 (3.70)	3 (4.84)	0 (0.00)	0.861	21 (3.80)	9 (3.72)	1 (2.94)	0.992
	**Ⅱ**	305 (61.24)	172 (62.77)	32 (60.38)		464 (61.38)	40 (64.52)	5 (71.43)		342 (61.96)	149 (61.57)	20 (58.82)	
	**Ⅲ**	173 (34.74)	94 (34.31)	18 (33.96)		264 (34.92)	19 (30.64)	2 (28.57)		189 (34.24)	84 (34.71)	13 (38.24)	
**Total**		498 (100.00)	274 (100.00)	53 (100.00)		756 (100.00)	62 (100.00)	7 (100.00)		552 (100.00)	242 (100.00)	34 (100.00)	

**Table 7:** HR in different genotypes of *HOTAIR* SNPs in univariate Cox regression analysis.

Genotype	DFS		OS	
	HR(95%CI)	P-value	HR(95%CI)	P-value
rs920778				
AA	1.000	–	1.000	–
GA	1.646(1.141-2.375)	0.007	1.786(1.008-3.163)	0.047
GG	1.909(1.005-3.625)	0.048	1.209(0.364-4.014)	0.757
rs1899663				
AA	1.000	–	1.000	–
CA	0.768(0.346-1.703)	0.007	1.011(0.232-4.397)	0.989
CC	0.537(0.247-1.166)	0.062	0.892(0.214-3.724)	0.876
rs4759314				
AA	1.000	–	1.000	–
GA	1.850(1.078-3.173)	0.026	2.792(1.357-5.745)	0.007
GG	0.981(0.137-7.028)	0.985	<0.001(0–∞)	0.996

**Table 8:** HR in different genotypes of *HOTAIR* SNPs in multivariate Cox regression analysis.

Genotype	DFS		OS	
	HR(95%CI)	P-value	HR(95%CI)	P-value
rs920778				
AA	1.000	–	1.000	–
GA	1.480(1.024-2.139)	0.037	1.547(0.871-2.746)	0.138
GG	1.795(0.939-3.429)	0.077	1.133(0.340-3.780)	0.838
rs1899663				
AA	1.000	–	1.000	–
CA	0.819(0.365-1.835)	0.627	1.019(0.233-4.460)	0.980
CC	0.648(0.296-1.416)	0.276	1.091(0.260-4.570)	0.905
rs4759314				
AA	1.000	–	1.000	–
GA	2.076(1.206-3.571)	0.008	3.472(1.675-7.197)	0.001
GG	0.844(0.116-6.130)	0.867	<0.001(0–∞)	0.968

**Figure 1:**
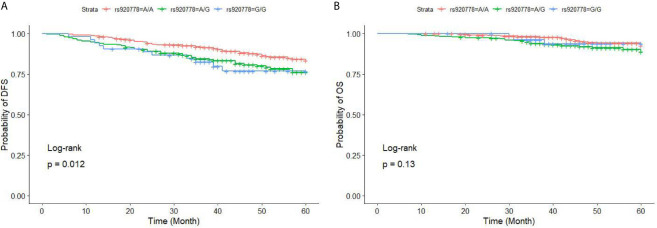
DFS **(A)** and OS **(B)** for BC patients with different genotypes of HOTAIR rs920778. BC, breast cancer; *HOTAIR*, HOX transcript antisense RNA; DFS, disease-free survival; OS, overall survival. The three curves of DFS are statistically significant (P = 0.012), subjects with GG genotype had a worst DFS (P = 0.048); the three curves of OS are not statistically different (P = 0.13), however, subjects with GA genotype had a worst OS than subjects with AA genotype (P = 0.047).

For the rs920778 SNP, there were many significant differences in DFS (P = 0.012) after comparing all three genotype of rs920778, the GG genotype was associated with the worst DFS of the three genotypes (GG, AG, and AA) in univariate analysis (HR = 1.909, P = 0.048). The AG genotype was associated with worse DFS than the AA genotype (HR = 1.48, P = 0.037). However, there was no significant difference in OS (P = 0.13). (**Figure 1** and **Table 8**).

**Figure 2:**
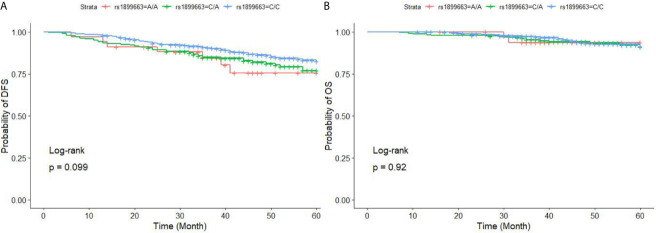
DFS **(A)** and OS **(B)** for BC patients with different genotypes of HOTAIR rs1899663. BC, breast cancer; *HOTAIR*, HOX transcript antisense RNA; DFS, disease-free survival; OS, overall survival. The three curves of DFS/OS are not statistically different, the P value is 0.099 and 0.92 respectively. However, subjects with CA genotype had a worse DFS compared to subjects with AA genotype (P = 0.007).

There was no difference in DFS or OS between individuals with the rs1899663 CC or CA genotypes and those with the AA genotype in multivariate analysis. (**Figure 2** and **Table 8**).

**Figure 3:**
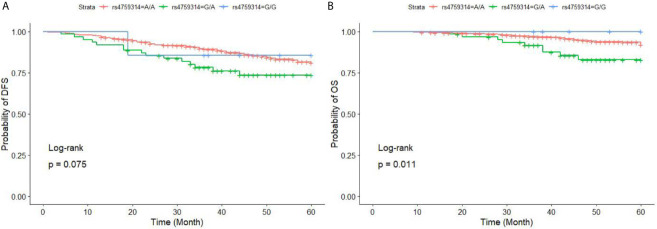
DFS **(A)** and OS **(B)** for BC patients with different genotypes of HOTAIR rs4759314. BC, breast cancer; *HOTAIR*, HOX transcript antisense RNA; DFS, disease-free survival; OS, overall survival. The overall three curves of DFS are insignificant different (P = 0.075), however, subjects with GA genotype had a worst DFS than subjects with AA genotype(P = 0.026); the overall three curves of OS are statistically significant, subjects with GA genotype had a worst OS in the three genotypes, however, subjects with GG genotype and AA genotype had a similar OS.

When comparing all three rs4759314 genotypes, the GA genotype had worse DFS and OS than those with the AA genotype (P = 0.008). The OS was significantly different when comparing all three genotypes (P = 0.011); individuals with the GA genotype had the worst OS(P=0.001). However, individuals with the GG genotype and those with the AA genotype had similar OS (P = 0.968) (**Figure 3** and **Table 8**).

## Discussion

*HOTAIR* is widely studied as an oncogene, and functional SNPs of *HOTAIR* have been related to cancer risk, including lung cancer, gastric cancer, esophageal cancer, cervical cancer and, prostate cancer, among others. Due to the difference in sample size and population characteristics, the relationship between the *HOTAIR* SNPs and BC risk is still contradictory. Our study may help to identify the significance of these three functional SNPs in BC susceptibility. Over-expression of *HOTAIR* is correlated with poor tumor prognosis, The expression of *HOTAIR* is regulated by multiple factors at the transcriptional and post-transcriptional levels, including estrogen receptors and estrogen receptor coregulators such as histone methylases MLL1 and MLL3 and CBP/p300 binding to the promoter of *HOTAIR* and regulating *HOTAIR* expression (18) and Pumilio homolog 1 regulating *HOTAIR* expression *via* a post-transcriptional mechanism (19). Three functional SNPs of *HOTAIR* can regulate *HOTAIR* expression (20–22), which may influence the BC prognosis. Our present study explored the relationship between the *HOTAIR* SNPs and BC prognosis.

The rs920778 SNP (G > A) is located in the intronic enhancer region of *HOTAIR*, and the AA genotype can increase the expression of *HOTAIR*. In our study, this SNP increased BC risk, which is consistent with the results of Bayram et al. (23), Rajagopal et al. (24), and Hassanzarei et al. (25) (**Table 9**). However, Yan et al. (26) found that the A allele is the most common genotype in the central Chinese population and could increase BC risk, which is contrary to the findings for northeast Chinese, southeast Iranian, South Indian, and Turkish populations (the present study, Hassanzarei et al.’s study, Rajagopal et al.’s study, and Bayram et al.’s study, respectively). We found that the G allele is rare and can increase BC risk. The distributions of rs920778 genotypes in BC patients in these five BC studies differ slightly. However, in these five BC studies, the AA genotype is more common whereas the GG genotype is rare. They differ from the distributions observed in other tumor studies [Yan et al. (26) found that the GG genotype is more common, the AA genotype is rare, and the A allele carries disease risk]. One possible reason for this is differences in tumor type and gender. Further, the study by Yan et al. has limitations in terms of sample size, detection methods, research results, and population. Therefore, we think that the rs920778 GG/AG genotypes can increase BC risk.

**Table 9:** Comparison of previous studies with our study in the association of HOTAIR SNPs and BC risk.

HOTAIR SNPs	Ethnicity	Source of Control	No. of Cases	Assay methods	Genotype			Genetic model	OR (P value)
Author rs920778					CC%	CT%	TT%		
Bayram et al. (23)	Turkish	Hospital^a^	123	TaqManSNP Genotyping	25.2	42.3	32.5	Recessive^a^	2.4 P=0.01
Yan et al. (26)	Chinese	Population	502	PCR-RFLP CRS–RFLP	2.4	30.1	67.5	T Allele	1.41 P=0.02
Hassanzarei et al. (25)	Southeast Iranian	Population	220	PCR-RFLP	15.0	54.1	30.9	Dominant^a^	2.64 P<0.0001
Rajagopal et al. (24)	Indian	Population	502	PCR-RFLP	17.3	50.2	32.5	Over-dominant^a^	1.31 P=0.031
Present Study 2021	Chinese	Population	828	MassAray system	6.4	33.2	60.4	Codominant^a^	2.426 P<0.001
rs1899663					GG%	GT&	TT%		
Yan et al. (26)	Chinese	Population	502	PCR-RFLP CRS–RFLP	67.53	31.35	3.97	T Allele	0.88 P=0.25
Hassanzarei et al. (25)	Southeast Iranian	Population	220	PCR-RFLP	37.7	55.0	7.3	Over-dominant^a^	0.38 P<0.0001
Khorshidi et al. (28)	Iranian	Population	122	ARMS-PCR	30.0	52.0	18.0		1.433 P=0.118
Lin et al. (21)	southeast Chinese	Population	969	PCR-RFLP	82.7	16.2	0.01		2.08 P=0.027
Rajagopal et al. (24)	Indian	Population	502	PCR-RFLP	38.5	45.4	16.1	dominant^a^	1.32 P=0.03
Present Study 2021	Chinese	Population	828	MassAray system	66.67	29.23	4.1	Recessive^a^	1.633 P=0.087
**rs4759314**					**AA%**	**AG%**	**GG%**		
Yan et al. (26)	Chinese	Population	502	PCR-RFLP CRS–RFLP	89.84	10.71	0.40	G Allele	0.9 P=0.57
Hassanzarei et al. (25)	Southeast Iranian	Population	220	PCR-RFLP	93.2	6.8	0	Codominant^a^	2.31 P=0.0808
Khorshidi et al. (28)	Iranian	Population	122	ARMS-PCR	79.0	21.0	1,0		0.755 P=0.316
Lin et al. (21)	southeast Chinese	Population	969	PCR-RFLP	82.7	16.2	0.01		1.12 P=0.52
Present Study 2021	Chinese	Population	828	MassAray system	91.64	7.52	0.84	overdominant^a^	0.566 P=0.001

^a^optimal model.

The rs1899663 SNP (C > A) is located in the intronic region of *HOTAIR*, and the AA genotype can increase the expression of *HOTAIR* by altering the binding affinity of various transcription factors, such as paired box 4, spermatogenic leucine zipper 1, and zinc finger protein 281 (ZFP281) (28) to *HOTAIR*. The results of studies on the relationship between the rs1899663 SNP and BC susceptibility remain controversial. It has been observed that rs1899663 polymorphism is associated with BC risk in the South Indian population (Rajagopal et al.’s study), the southeast Chinese population (Lin et al.’s study) (21), and the Southeast Iranian population (Hassanzarei et al.’s study) (**Table 9**). However, no relationships were observed in the central Chinese population (Yan et al.’s study), northeastern Chinese population (the present study), or in the Iranian population (Khorshidi et al.’s study) (27). Two smaller studies from Iran (Hassanzarei et al. and Khorshidi et al.) have inconsistent results, as do three larger Chinese studies (Lin et al., Yan et al., and the present study). In Taheri et al.’s study, the relationship between rs1899663 SNP and prostate cancer susceptibility was not observed due to the sample size, however, they compared prostate hyperplasia tissues and prostate cancer tissues and identified that the risk of AA alleles in tumor tissues was higher than CC alleles, This result suggests that AA alleles might increase prostate cancer susceptibility (28).The P value of 0.087 in the present study is close to 0.05. Therefore, we think that SNP has a weak relationship with BC risk when increasing the sample size due to the weak effect of rs1899663 SNP on BC risk.

The rs4759314 SNP (A > G) is located in intronic region of *HOTAIR*, and the GG genotype can increase the expression of *HOTAIR* by enhancing the promoter activity of *HOXC11*. Of five studies examining the relationship between rs4759314 and BC susceptibility (**Table 9**), only two Chinese studies [Yan et al. (26) and this study] have shown a significantly decreased risk of BC in individuals with at least one G allele (GA or GG) compared to individuals with homozygous A alleles. The other three studies did not show any association of rs4759314 with BC risk. Two studies in the Iranian population [Hassanzarei et al (25). and Khorshidi et al. (27)] are too small to draw such conclusions, and another Chinese study in southeast China (Lin et al.’s study) showed that rs4759314 has no correlation with the risk of BC (21). This may be because BC has a population bias, and the population in the other two studies are in middle and northeast China.

We also examined the haplotypes of these three SNPs. We found that the rs920778–rs1899663 and rs920778–rs1899663–rs4759314 haplotypes significantly increase BC risk (P < 0.001). We believe that the gene effect of rs920778 affects the gene effects of the other two SNPs, which leads to an increase in breast cancer susceptibility.

In Bayram’s study, researchers found an association between the rs920778 SNP and clinicopathological features in the Turkish population, including advanced TNM stage, larger tumor size, distant metastasis, perineural invasion, and poor histological grade (23). In Hassanzarei’s study, they found that the rs920778 SNP was only significantly associated with ER status (25). In Rajagopal’s study, they found that the rs920778 variant (AG + GG genotype) increased BC risk in premenopausal women (OR = 5.86, 95% CI = 3.87–8.88, P < 0.0001) (24). However, we did not find any relationship between the rs920778 SNP and any clinicopathological features. This may be because all of these studies were retrospective and there might be an inherent selection bias. Because of the low distribution frequency of the GG genotype (about 3–8% among common populations), a large sample size is needed to analyze the relationship between the GG genotype and clinical characteristics.

We initially found that the rs920778 SNP is associated with the prognosis of BC patients. Our study found that the DFS of patients with the AG/GG genotypes was much shorter than that of patients with the AA genotype (P = 0.012). However, we did not find similar results for OS. Our result is consistent with the result of Weng et al’s (29) study showing that subjects with GG genotype of rs920778 had a poor OS, however Xavier-Magalhhães et al’s study (30) had the opposite result that subjects with the AG genotype of rs920778 had a longer overall survival than GG subjects in glioma patients. The sample size and tumor type might result the inconsistent results. *HOTAIR* is regarded as an oncogene involved in both the initiation and progression of cancer. The rs920778 SNP is located in the intronic enhancer region of *HOTAIR*, and polymorphism of rs920778 could alter the activity of this enhancer and lead to overexpression of *HOTAIR*. Elevated expression of *HOTAIR* has been reported to be associated with reduced DFS and OS in cervical cancer patients (31). Therefore, we infer that the influence of the rs920778 SNP on BC prognosis is mediated by the resultant increased expression of *HOTAIR*. We need to prove this hypothesis further in BC tissue.

The rs1899663 SNP had no effect on DFS. However, in subgroup analysis, individuals with the CA genotype had worse DFS than those with the AA genotype (P = 0.007), which could provide references for future research. Individuals with the rs4759314 GA genotype had worse DFS and OS than patients with other genotypes(P=0.008 and P=0.001 respectively), which was also interesting and needed further study. Because of the low distribution frequency of the rare genotypes AA of rs1899663 and GG of rs4759314 (no more than 2.4%), a larger sample size is needed to assess their associations with prognosis. Because the rs1899663 and rs4759314 SNPs can increase the expression of *HOTAIR*, their effect on BC prognosis appears to be mediated by the increased expression of *HOTAIR*. However, we need to prove this hypothesis further in BC tissue. Although all the results of survival analysis have not been verified in multivariate analysis, our results suggest that some gene loci may play a role in the occurrence and development of BC.

In summary, this study demonstrates, for the first time, that functional *HOTAIR* SNPs rs920778 and rs4759314 are related to the risk and prognosis of BC in the northeastern Chinese population, suggesting that these two SNP sites may be involved in the occurrence, development, and metastasis of BC by regulating the expression of *HOTAIR*. This may have certain significance for future diagnosis, drug development, and prognostic judgment of BC. The distribution of gene frequency of the three functional *HOTAIR* SNP loci has a certain correlation with regions and populations. This study only examined the northeast Chinese population as its research object, and it therefore cannot explain why these three *HOTAIR* SNP loci are responsible for the occurrence and development of BC in the overall Chinese population. Therefore, we need a more large prospective multi-center, multi-regional, multi-ethnic population to analyze the significancy of *HOTAIR* SNP in BC development and find a target of treatment.

## Data Availability Statement

The original contributions presented in the study are included in the article/supplementary files, further inquiries can be directed to the corresponding authors.

## Ethics Statement

The studies involving human participants were reviewed and approved by Institutional Ethics Committee of our hospital (ethical approval number 2014-031). The patients/participants provided their written informed consent to participate in this study.

## Author Contributions

JC and MY conceived and designed the experiment. NC, LJ, and RB performed the experiment. CK analyzed the data. SX and GY collected clinical information. ZL wrote the manuscript. All authors contributed to the article and approved the submitted version.

## Funding

This study was financially supported by the Natural Science Foundation of Jilin Province (20200201474JC).

## Conflict of Interest

The authors declare that the research was conducted in the absence of any commercial or financial relationships that could be construed as a potential conflict of interest.

## Abbreviations

BC, breast cancer; HOTAIR, HOX transcript antisense RNA; SNP, single nucleotide polymorphism; OR, odds ratio; CI, confidence interval; DFS, disease-free survival; OS, overall survival.
